# Baicalin positively regulates osteoclast function by activating MAPK/Mitf signalling

**DOI:** 10.1111/jcmm.13066

**Published:** 2017-02-03

**Authors:** Li Lu, Li Rao, Huanhuan Jia, Jun Chen, Xingyan Lu, Guozhu Yang, Qingnan Li, Kenneth Ka Ho Lee, Li Yang

**Affiliations:** ^1^ School of Life Science and BiopharmacyGuangdong Key Laboratory of Pharmaceutical Bioactive SubstancesGuangdong Pharmaceutical UniversityGuangzhouChina; ^2^ Guangdong Key Laboratory of Laboratory AnimalsGuangdong Laboratory Animals Monitoring InstituteGuangzhouChina; ^3^ Stem Cell and Regeneration Thematic Research ProgramSchool of Biomedical SciencesThe Chinese University of Hong KongShatinHong Kong SARChina; ^4^ Zhujiang Hospital of Southern Medical UniversityGuangzhouChina

**Keywords:** baicalin, osteoclast, MAPK/Mitf signalling, bone fracture healing

## Abstract

Activation of osteoblasts in bone formation and osteoclasts in bone resorption is important during the bone fracture healing process. There has been a long interest in identifying and developing a natural therapy for bone fracture healing. In this study, we investigated the regulation of osteoclast differentiation by baicalin, which is a natural molecule extracted from *Eucommiaulmoides* (small tree native to China). It was determined that baicalin enhanced osteoclast maturation and bone resorption activity in a dose‐dependent manner. Moreover, this involves the activation of MAPK, increased Mitf nuclear translocation and up‐regulation of downstream osteoclast‐related target genes expression. The baicalin‐induced effect on osteoclast differentiation can be mimicked by specific inhibitors of p‐ERK (U0126) and the Mitf‐specific siRNA, respectively. Protein–ligand docking prediction identified that baicalin might bind to RANK, which is the upstream receptor of p‐ERK/Mitf signalling in osteoclasts. This indicated that RANK might be the binding target of baicalin. In sum, our findings revealed baicalin increased osteoclast maturation and function *via* p‐ERK/Mitf signalling. In addition, the results suggest that baicalin can potentially be used as a natural product for the treatment of bone fracture.

## Introduction

Osteoclasts are multinucleated cells of hematopoietic origin that play key roles in the regulation of bone mass, as well as bone quality. The development of osteoclasts is essential for meeting the structural and metabolic demands of the skeleton which are subjected to constant mechanical changes, and nutritional demands, especially during bone fracture healing. Recently, it has been reported that osteoclasts are active from the very early phases of bone fracture healing and remain active over the entire course 1. Moreover, the study emphasizes the important role of osteoclasts during the entire course of fracture healing. These osteoclasts apparently alter function between a pure resorption activity (to remove redundant mineralized callus tissue) and an active remodelling role and in cooperation with osteoblasts optimize the bone structure and alignment. It appears that the state of osteoclastic activity is dependent on the fracture healing phase, as well as on the localization of the cells within the callus. It indicates osteoclasts are active than usual in the stage of bone fracture. Hence, therapeutic drugs designed for improving bone fracture healing should take into account on when both osteoclasts and osteoblasts are most active.

It has been demonstrated that microphthalmia‐associated transcription factor (Mitf) is required for the proper development of several cell lineages that includes osteoclasts, melanocytes, retinal pigment epithelial cells, mast cells and natural killer cells 2. Mouse model studies have indicated a critical role for Mitf in regulating osteoclast development. Mutations of Mitf in different organisms have resulted in development of osteopetrosis due to defective osteoclast development. Mitf has been shown to be a target of both the macrophage‐colony stimulating factor (M‐CSF) 3 and receptor activator of nuclear factor kappa B ligand (RANKL) 4 signalling pathways in osteoclasts. During the osteoclast development process, osteoclasts are stimulated by M‐CSF and RANKL that initiate a cascade of events leading to the activation of Mitf *via* the phosphor‐ERK (p‐ERK) 3 and phosphor‐p38 (p‐p38) 5. Sharma *et al*. 6 indicated that the Mitf complex integrated the signals necessary for the appropriate temporal expression of osteoclast‐associated genes such as cathepsin K (CtsK) and acid phosphatase 5 (Acp5) during osteoclast differentiation. Furthermore, it has been demonstrated that Mitf acts synergistically with PU.1 and nuclear factor of activated T cells cytoplasmic 1 (NFATc1) to activate Acp5, CtsK and osteoclast‐associated receptor (OSCAR) promoters during osteoclast differentiation 6, 7.

Baicalin is a natural molecule found in the roots of the Baical skullcap (*Scutellaria baicalensis Georgi*) 8. It is also one of the major active components found in ‘DuZhong’ (*Eucommiaulmoides*) that is commonly used for promoting bone fracture healing in Chinese traditional medicine 9. Baicalin has attracted considerable attention because it has a variety of interesting properties such as antibacterial 10, antiviral 11, 12, antitumour 8, 9 and anti‐inflammatory effects 13, 14. A recent study has shown that baicalin, but not baicalin, can enhance osteoblast bone formation activities *via* the Wnt/β‐catenin signalling pathway 15—but little is known about its effect on osteoclast bone resorption. Since ‘DuZhong’ is often prescribed for use in bone fracture healing in Chinese traditional medicine, the healing may be improved by baicalin. We hypothesize that baicalin can exert an effect not only on active osteoblasts but also on osteoclasts during bone fracture healing. In the present study, we determined that baicalin exerts a positive effect on osteoclast maturation and resorption functions. We also investigated the mechanisms of baicalin's action on osteoclasts by examining the p‐ERK/Mitf signalling pathway. Here, we have shown that baicalin promoted osteoclast maturation and function by activating p‐ERK and increasing nuclear translocation of Mitf, with RANK a potential target of baicalin.

## Materials and methods

### Reagents

Baicalin was purchased from Sigma‐Aldrich (St. Louis, MO, USA). Recombinant murine M‐CSF and RANKL were purchased from Peprotech (Rocky Hill, NJ, USA). Anti‐Mitf, anti‐ERK, anti‐phospho ERK, anti‐p38, anti‐phospho p38 and anti‐β**‐**actin antibodies were all purchased from Cell Signaling Technology, Inc. (Danvers, MA, USA). Anti‐MMP9 antibody was purchased from Abcam, Inc. (dilution 1:1000; Danvers, MA, USA). α‐Modified essential medium (α‐MEM) and rhodamine phalloidin were obtained from Life Technologies Corp. (Carlsbad, CA, USA), and TRAP staining kit was purchased from Sigma‐Aldrich. Mounting medium was purchased from Vector Laboratories, Inc. (Burlingame, CA, USA). Cell Counting Kit‐8 was purchased from Dojindo Molecular Technologies (Dojindo, Tokyo, Japan). All other chemicals were obtained from Sigma‐Aldrich.

### Cell culture and osteoclast induction

Mouse monocyte macrophage RAW264.7 cells were maintained in α‐MEM supplemented with 10% foetal bovine serum (FBS), 100 U/ml of penicillin and 100 mg/ml streptomycin. The medium was changed every 3 days and cells cultured in humidified atmosphere of 5% CO_2_ at 37°C. Primary mouse bone marrow‐derived macrophages (BMMs) were used for osteoclast differentiation. For generation of bone marrow‐derived osteoclasts, monocytes were isolated from femur and tibiae of C57B/6 mice (Central Lab. Animal Inc., Guangzhou, China), seeded and cultured in α‐MEM plus 10% FBS and 10 ng/ml M‐CSF (Peprotech, Inc.) overnight. Suspended cells were used as osteoclasts precursors. Induction of BMMs to differentiate into osteoclasts was achieved by seeding those cells into a 24‐well plate at the density of 8 × 10^5^ cells/well in α‐MEM with 10% FBS, 100 ng/ml RANKL (Peprotech, Inc.) and 25 ng/ml M‐CSF. Multinucleated osteoclasts were observed on differentiation day 4–6.

### Cell viability assay

RAW264.7 cells and BMMs were plated onto 96‐well plates in α‐MEM containing 10% FBS, at the densities of 1 × 10^3^ and 1 × 10^4^ cells/well, respectively. After 24 hrs, the cultures were treated with serially diluted compounds and incubated for 1 or 3 days. Cell viability was then measured by Cell Counting Kit‐8 according to the manufacturer's protocol. The experiment was performed in triplicates.

### TRAP activity assay

For measuring TRAP activity, cells were fixed in 10% formalin for 10 min., rinsed in PBS for 3 changes and stained using a Leukocyte Acid Phosphatase kit (Sigma‐Aldrich). Images of TRAP‐positive cells were captured under a microscope with a DP Controller (Olympus Optical, Tokyo, Japan). The number of mature osteoclasts was quantified by counting the number of multinucleated TRAP^+^ cells (>3 nuclei) in a representative area, in each of the three replicate samples.

### Immunofluorescent staining

Cells were washed with PBS twice, fixed with 10% formalin for 10 min., permeabilized with ice cold acetone for 5 min. and washed twice in PBS. For Mitf immunofluorescent staining, cells were first incubated in blocking solution (5% non‐fat dry milk in TBS containing 0.1% Tween 20) for 1 hr to reduce non‐specific binding. Then, the samples were exposed to primary antibodies overnight at 4°C, washed three times in PBS and incubated with secondary goat anti‐rabbit fluorescent antibody for 1 hr. Actin fibres were then stained with 20 μM rhodamine phalloidin (Sigma‐Aldrich) for 20 min. at room temperature and protected against light. After washing in PBS twice, the samples were treated with mounting medium containing DAPI for 5 min. and covered using glass coverslips. The immunofluorescent staining was viewed and images captured using an Olympus FV1000 confocal microscope.

### *In vitro* bone resorption assays

Bone marrow‐derived macrophages (5 × 10^4^ cells/well) were seeded onto bovine cortical bone slices placed in 24‐well culture plates, along with M‐CSF (25 ng/ml) and RANKL (100 ng/ml), and treated under conditions indicated in individual experiments. Bone slices were then harvested and cells were removed with 0.25 m ammonium hydroxide and mechanical agitation. Bone resorption pits were visualized and imaged by scanning electron microscopy using a Philips 515 scanning electron microscope (SEM). Bone resorption assays were performed in triplicates, and a representative area from each assay was displayed. The data were quantified by measuring the percentage of resorbed areas in three randomly selected resorption areas. The percentage of the resorbed area was determined using a Metamorph analysis system.

### RNA isolation and gene expression analysis

Total RNAs were extracted from cells using TRIzol reagent (Invitrogen, Carlsbad, CA, USA), and 1 μg of total RNA were used for qRT‐PCR analyses. RNA was reverse‐transcribed using a First Strand cDNA Synthesis kit (Fermentas, Hanover, MD, USA). qRT‐PCRs were performed in triplicate using the 7900 fast real**‐**time PCR system (Applied Biosystems, Foster City, CA, USA) and detection system following the manufacturer's protocols. Expressions of the gene of interest were normalized relative to levels of β**‐**actin expression.

### Western blotting

After cells had been washed and lysed, the cell lysates were boiled in SDS sample buffer and subjected to electrophoresis on 8% SDS‐PAGE. Proteins were transferred onto nitrocellulose membranes using a semi‐dry blotter (Bio‐Rad Laboratories, Hercules, CA, USA) and incubated in blocking solution (5% non‐fat dry milk in TBS containing 0.1% Tween 20) for 1 hr to reduce non‐specific binding. Membranes were then exposed to primary antibodies overnight at 4°C, washed three times and incubated with appropriate secondary goat antimouse or anti‐rabbit antibodies for 1 hr. Membranes were washed extensively and enhanced for chemiluminescence detection assay performed according to the manufacturer's directions.

### Ligand–protein docking prediction

The ligand–protein docking prediction was performed using the SwissDock system, which is a web provider dedicated service for the small molecules and target proteins docking prediction. To submit the docking task, we selected the RANK as target protein. Then, we searched the baicalin structure in the database and selected the proper structure for docking task. All docking calculations were completed on the web side. The docking results were demonstrated in the UCSF Chimera molecular viewer which can be used for result analysis 16.

### Statistical analysis

All statistical analyses were performed using a SPSS 5.0 software (IBM, Armonk, NY, USA) and one‐way anova;* P* < 0.05 was considered to be statistically significant.

## Results

### Baicalin promotes osteoclast maturation and bone resorption activity but not cell proliferation

**Figure 1:**
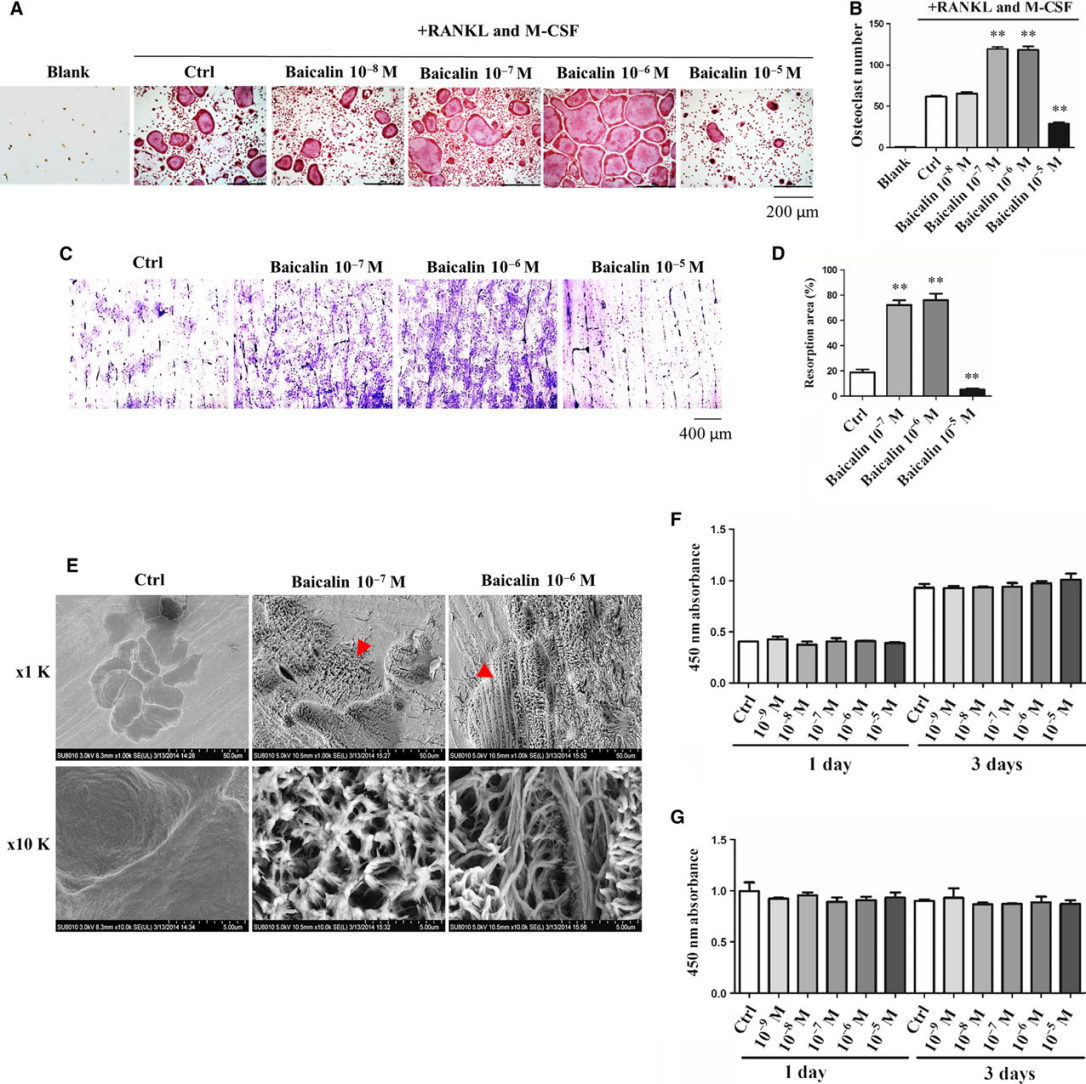
Baicalin enhanced RANKL‐induced osteoclast maturation and bone resorption activity, but did not affect cell viability of RAW264.7 and BMM cells. (**A**) BMMs were cultured with or without 100 ng/ml RANKL and 25 ng/ml M‐CSF for 3 days and then treated with and without baicalin for 3 days. (**B**) TRAP^+^ multinucleated (more than three nuclei) cells were counted. **P < 0.001 (*n* = 3). (**C**) Bone‐resorbing activity assay, where BMMs were introduced into bone slices and induced by RANKL and M‐CSF for 4–6 days. After confirmation of osteoclast maturation, the osteoclasts on the bone slices were treated with or without baicalin for 7 days. Resorption pits were stained by toluidine blue; (**D**) resorption pits’ area was quantified using a Metamorph analysis system. **P < 0.001 (*n* = 3). (**E**) Resorption pits were scanned by electron microscope. The red triangles indicate the collagen bundles in the baicalin treatment group but not in the control group. (**F** and **G**) shows the effect of baicalin on RAW264.7 and BMMs cell viability.

The effect of baicalin on the osteoclast differentiation was evaluated in BMM cultures induced by RANKL and M‐CSF. Baicalin was found to significantly promote the transformation of BMMs into TRAP^+^ multinucleated osteoclasts, in a dose‐dependent manner, as compared with the control group (Fig. 1A and B). The most effective dosage of baicalin was determined to be 10^−6^M. However, the number of TRAP^+^ osteoclasts was significantly decreased in the presence of 10^−5^M baicalin as compared with the control group. To assess the effect of baicalin in bone resorption, BMMs were introduced into bone slices and induced with M‐CSF and RANKL. After osteoclast maturation was confirmed, the bone‐resorbing activity was assessed with or without baicalin (10^−7^–10^−5^M) treatments. Toluidine blue staining results revealed that 10^−7^M and 10^−6^M baicalin enhanced osteoclast bone resorption activities as indicated by the significant increase in the appearance of bone resorption areas (Fig. 1C and D). These results are consistent with the TRAP staining results which shows that 10^−5^M baicalin inhibited osteoclast formation. The SEM images revealed that there were more collagen structures exposed in the baicalin treatment group. This is reflected by the marked increase in the deepness of the resorption pits in the 10^−7^ and 10^−6^M baicalin treatment groups as compared with control group (Fig. 1E). Interestingly, treatment of RAW264.7 cells and BMMs with baicalin for 1–3 days did not affect cell proliferation (Fig. 1F) and cell viability (Fig. 1G), respectively.

### Baicalin enhances the actin ring formation and osteoclastogenesis genes expression

**Figure 2:**
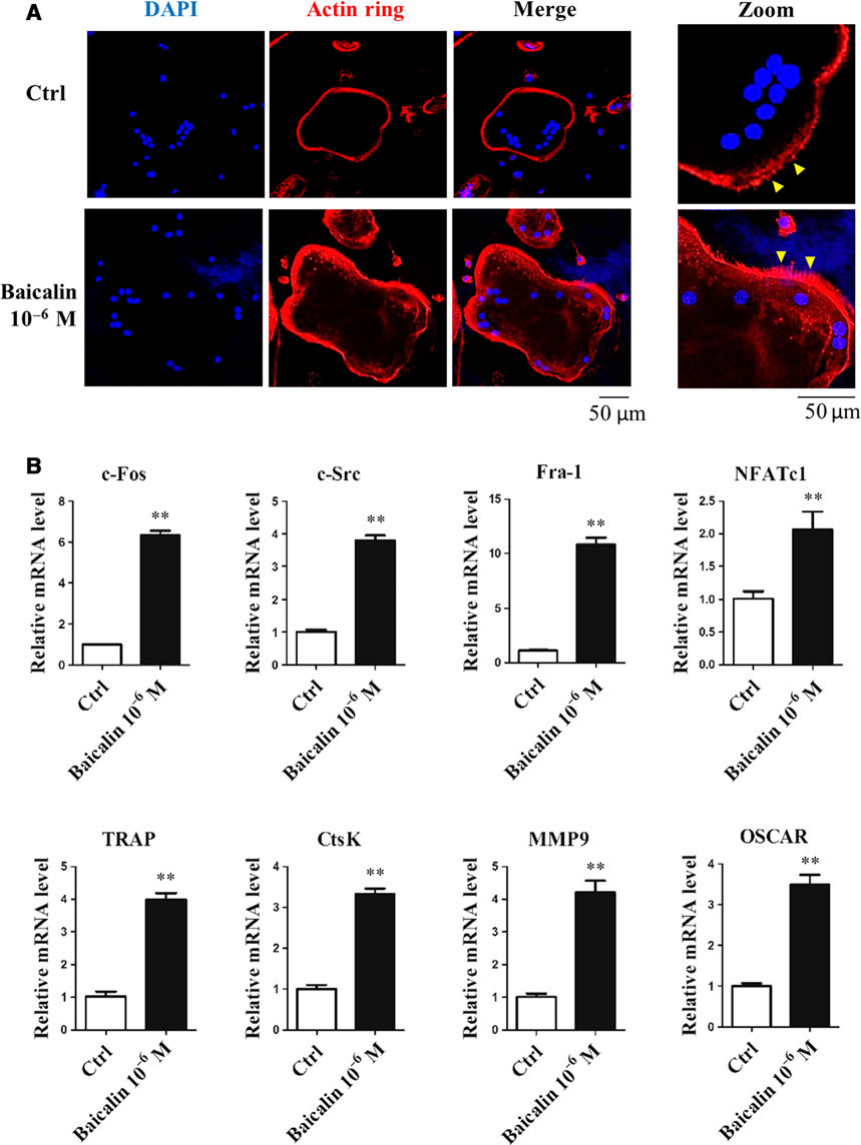
Baicalin promoted osteoclast actin ring formation and enhanced osteoclastogenesis‐associated genes expression. (**A**) In the actin ring formation assay, BMMs were plated into 24‐well plates and cultured with RANKL and M‐CSF for 3 days and then cultured in the absence or presence of baicalin for 24 hrs. Cells were fixed and stained use fluorescein rhodamine phalloidin to detect the presence of actin rings. The yellow triangles indicate the ruffled edge of the actin ring. (**B**) RAW264.7 cells were plated into 6‐well plate and then cultured in the absence or presence of baicalin for 3 days. Baicalin up‐regulated expression of osteoclastogenesis‐associated genes (c‐Fos, c‐Src, Fra‐1, TRAP, CtsK, MMP9 and OSCAR). **P < 0.001 (*n* = 3).

We conducted immunofluorescent staining to evaluate actin ring formation in the mature osteoclasts under the influence of baicalin. The presence of an actin ring structure is considered to be a characteristic feature of mature osteoclast 17. The ring is important for osteoclasts to attach to the active bone surface during bone resorption. Immunofluorescent staining for the actin ring in osteoclasts demonstrated that baicalin treatment produced a stronger fluorescence signal (red) and a more define ruffled membrane edge (yellow triangles) compared with non‐treated osteoclasts (Fig. 2A). We also quantitated the expression of genes associated with osteoclast differentiation and maturation‐associated genes, specifically four transcription factors: c‐Fos, c‐Src, Fra‐1 and NFATc1, and four special marker genes of mature osteoclast: TRAP, CtsK, MMP9, and OSCAR (Fig. 2B). The results revealed that gene expressions associated with osteoclastogenesis were significantly increased in the baicalin treatment group compared with the control group.

### Mitf and MAP kinases play a role in the induction of BMMs to osteoclasts by baicalin

**Figure 3:**
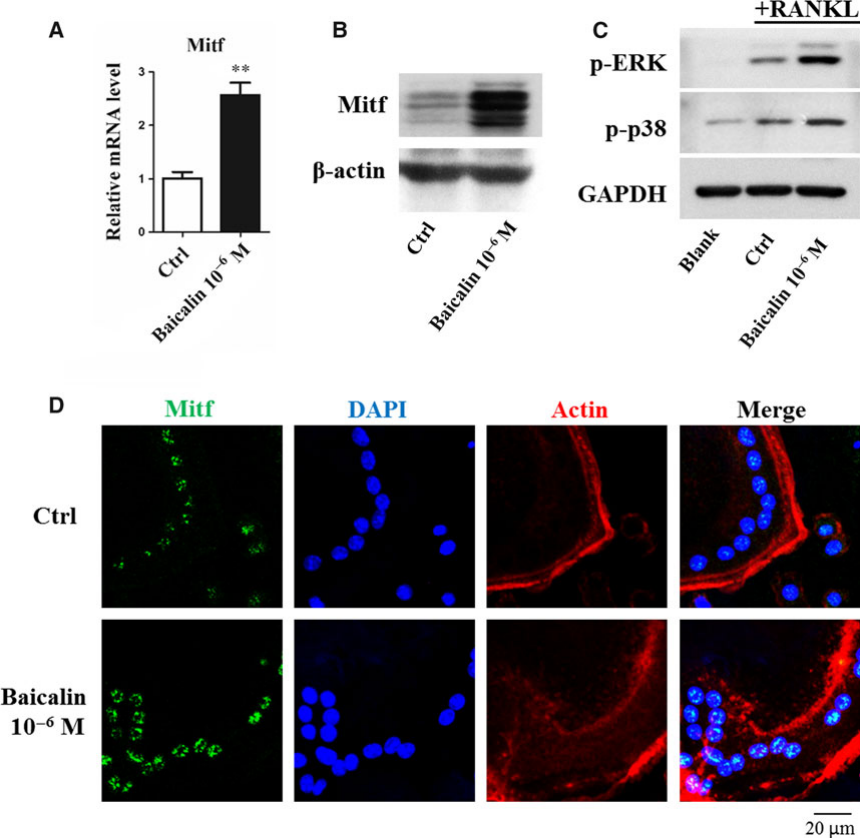
Mitf and MAP kinases play a role in the induction of osteoclasts by baicalin. (**A** and **B**) Baicalin increased Mitf expression in mature osteoclasts as determined by qRT‐PCR and Western Blot. **P < 0.001 (*n* = 3). (**C**) BMMs were plated into 24‐well plate and cultured with RANKL and M‐CSF for 3 days and then cultured in the absence or presence of baicalin for 24 hrs. Cells were fixed and immunofluorescently stained to detect the presence of Mitf translocation. Baicalin increased Mitf nuclear translocation in mature osteoclasts (*n* = 3). (**D**) RAW264.7 cells were plated into 6‐well plate and treated with baicalin for 2 hrs before RANKL treatment and protein samples were prepared 30 min. after RANKL treatment. p‐ERK and p‐p38 expressions were promoted in mature osteoclasts by baicalin as determined by Western Blot (*n* = 3).

We assessed the role of Mitf in osteoclast differentiation and function by measuring its mRNA and protein levels following baicalin treatment. qRT‐PCR and Western blot analysis revealed that both mRNA and protein levels for Mitf were significantly up‐regulated by baicalin (Fig. 3A and B). Mitf has been reported to be a key transcription factor during the osteoclast differentiation and maturation. To further confirm that the effect of baicalin was mediated by Mitf, we performed immunofluorescent staining to determine whether Mitf nuclear translocation was affected by baicalin. Immunofluorescent staining revealed baicalin markedly enhanced the translocation of Mitf into the nucleus of mature osteoclasts, as compared with the control (Fig. 3C). The MAP kinases have been reported as the upstream regulators of Mitf, so Western blot was employed to detect MAP kinase activation. The blots demonstrated an increase in p‐ERK and p‐p38 expression in baicalin‐treated cells as compared with the blank group (Fig. 3D).

### U0126 inhibits the baicalin‐induced effect on osteoclasts

**Figure 4:**
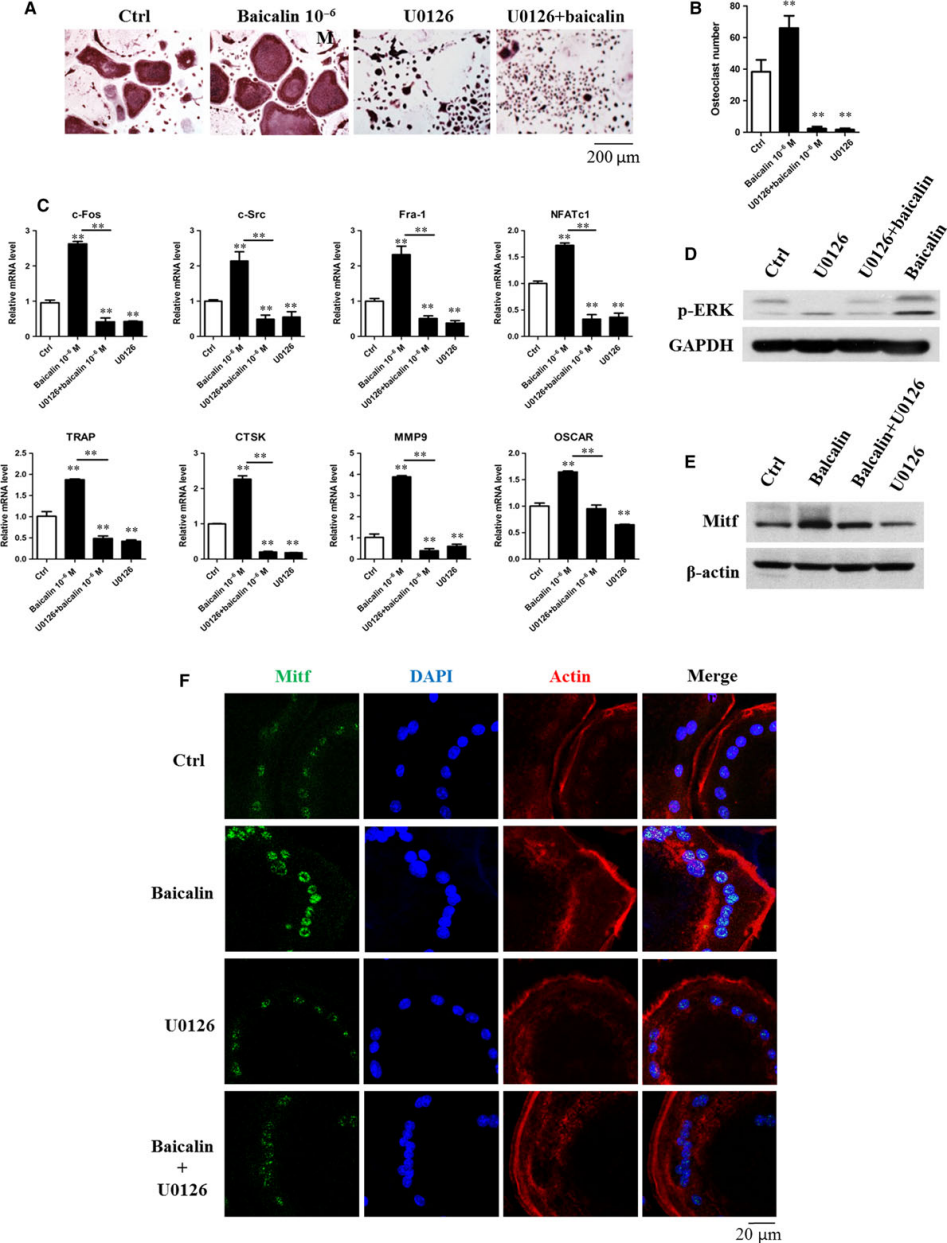
U0126 inhibited baicalin‐induced bioactivity in osteoclasts by blocking p‐ERK/Mitf signalling. (**A**) BMMs were cultured with or without 100 ng/ml RANKL and 25 ng/ml M‐CSF in 24 well for 3 days and then in the absence or presence of baicalin or U0126 for 3 days. (**B**) Numbers of TRAP^+^ multinucleated cells were counted. **P < 0.001 (*n* = 3). (**C**) qRT‐PCR shows U0126 suppressed the enhancing effect of baicalin on osteoclastogenesis‐associated genes expression by RAW264.7 cells. **P < 0.001 (*n* = 3). (**D** and **E**) RAW264.7 cells were treated with U0126 and/or baicalin for 2 hrs followed by RANKL treatment for 30 min. Western blot revealed U0126 inhibited p‐ERK and Mitf expression (*n* = 3). (**F**) Mitf nuclear translocation in mature osteoclast was detected by immunofluorescent staining. BMMs were cultured with or without 100 ng/ml RANKL and 25 ng/ml M‐CSF for 3 days, and then in the absence and presence of baicalin or U0126 for 1 day (*n* = 3).

We investigated whether the MAPK/Mitf signalling pathway participated in the effect of baicalin on osteoclasts. U0126, a small molecule inhibitor of p‐ERK, was introduced into the *in vitro* cultures. As shown in Figure 4A and B, baicalin increased the number of TRAP^+^ osteoclasts while U0126 exposure markedly inhibited this bioactivity. This observation was further supported by qRT‐PCR analysis that expressions of all the osteoclastogenesis genes were suppressed by U0126 (Fig. 4C). The enhancing effect of baicalin on p‐ERK and Mitf was significantly inhibited by U0126 (Fig. 4D and E). Immunofluorescent staining showed that Mitf nuclear translocation was markedly reduced when baicalin and U0126 were used in combination (Fig. 4F). This suggests that the baicalin‐inducing effect on Mitf nuclear translocation can be eliminated by U0126, which accordingly suppress the baicalin‐inducing effect on osteoclast formation and function by inhibiting p‐ERK which act upstream of Mitf.

### Mitf‐specific siRNAs suppress baicalin‐induced effects on osteoclasts

**Figure 5:**
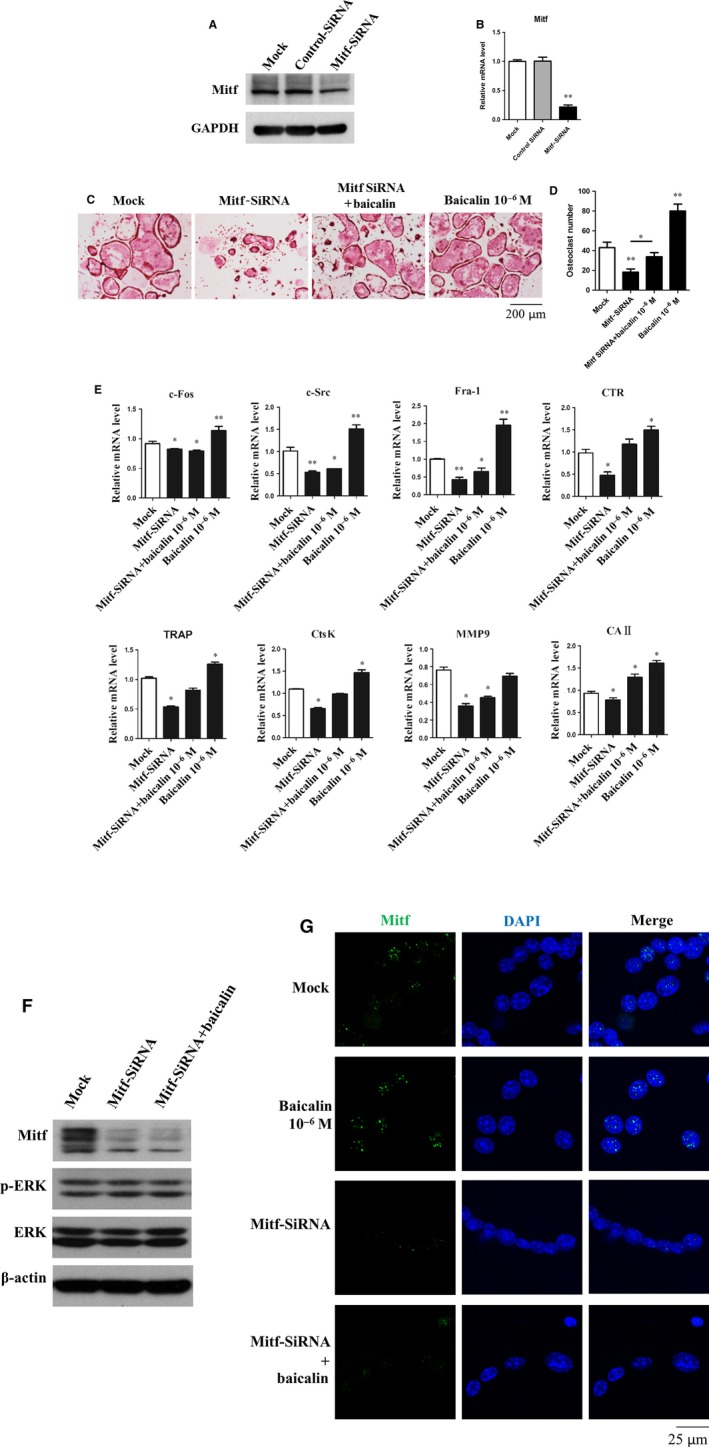
Baicalin‐induced osteoclast maturation and activity are mediated by Mitf. BMMs were cultured with or without 100 ng/ml RANKL plus 25 ng/ml M‐CSF for 3 days and then transfected with Mitf‐specific siRNAs for 2 days. The silencing of Mitf expression was validated by Western blot (**A**) and qRT‐PCR (**B**). (**C**) Mitf knockdown significantly inhibited osteoclast maturation. (**D**) Number of TRAP^+^ osteoclasts were determined following Mitf‐siRNA and baicalin treatment.*P <* *0.005 and **P <* *0.001 (*n *=* *3). (**E**) Silencing Mitf expression significantly down‐regulated c‐Fos, c‐Src, Fra‐1, NFATc1, TRAP, CtsK, MMP9 and OSCAR expression. **P <* *0.001 (*n *=* *3). (**F**) Western blot revealed Mitf knockdown did not affect p‐ERK and total ERK expression. Baicalin cannot increase Mitf expression when used with Mitf‐siRNA. (**G**) Mitf nuclear translocation in mature osteoclast was detected by immunofluorescent staining (*n *=* *3).

To acquire further insight into the regulation role of Mitf in baicalin‐induced osteoclast differentiation and function, we used siRNA to specifically silence Mitf expression (the siRNA knockdown efficiency is indicated in Fig. 5A and B). We first examined the effect of Mitf knockdown on osteoclast maturation using TRAP staining. We found that silencing Mitf expression significantly inhibited the formation of TRAP^+^ multinucleated osteoclasts, and baicalin could partially rescue the inhibitory effects of Mitf‐siRNA (Fig. 5C and D). This was further supported by qRT‐PCR analysis (Fig. 5E) that showed the down‐regulation of osteoclast markers upon Mitf knockdown. These findings suggest that enhancing effects of baicalin on gene expressions were abolished by silencing Mitf. Western blot showed that ERK expression and activation were not affected by Mitf knockdown (Fig. 5F). To further confirm the effects of Mitf‐siRNA on its nuclear translocation, we tracked the localization of Mitf under different conditions. Same as Figure 3C, like our immunofluorescent staining, there were more cells with Mitf nuclear translocation than in the control group, in the presence of baicalin. Silencing Mitf expression eliminated the inducing effect of baicalin on Mitf nuclear translocation (Fig. 5G).

### Protein binding prediction of baicalin and RANK

**Figure 6:**
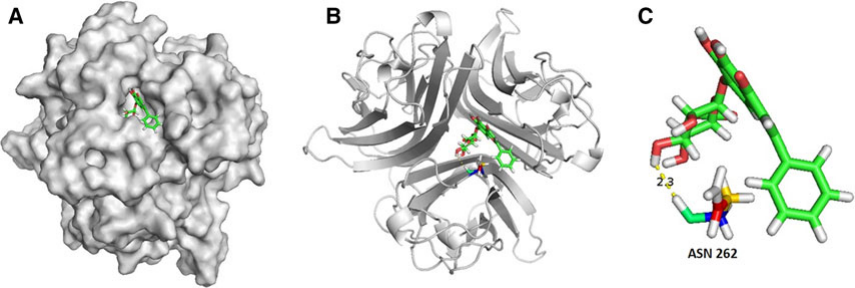
SwissDock ligand–protein docking prediction of baicalin and RANK (**A**) illustrates the fit docking of baicalin and RANK. (**B**) Baicalin predicted to bind with RANK on residue Asn 262, with binding energy ▵G = −12.63 kcal/mol. (**C**) The docked pose of Asn 262 with baicalin is shown. The distance between baicalin and RANK residue is 2.3 Å. The backbone of Asn 262 is predicted to form a hydrogen bond with baicalin.

Using SwissDock ligand–protein docking system, we predicted the baicalin binding site in RANK. The binding energy (▵G) of baicalin and RANK was −12.63 kcal/mol. The result demonstrated that baicalin might bind to RANK on the residues: Asparagine 262 (Asn 262). The distance between baicalin and Asn 262 is 2.3 Å (Fig. 6C). The finding is indicative of a hydrogen bond interaction between the protein and ligand.

## Discussion

Understanding the regulatory mechanisms involved in osteoclast differentiation is essential for comprehending the pathogenesis and development of a therapy for treating bone diseases, such as non‐union bone repair. It has been reported that baicalin stimulates osteoblast bone formation activity *via* the activation of the Wnt/β‐catenin signalling pathway 15. However, there have been few studies done on whether baicalin could also regulate osteoclast differentiation and bone resorption activity, and if it does, how does baicalin regulate osteoclast differentiation. Presently, we have elucidated the regulatory mechanism of baicalin in the control of cell differentiation and the promotion of bone‐resorbing activity in mature osteoclasts. Baicalin's bioactivity is trigged by the activation of MAP kinases and the nuclear translocation of Mitf which activate genes associated with osteoclastogenesis.

In our functional studies, we examined the effects of baicalin on osteoblasts by monitoring its TRAP^+^ multinuclear cell formation and bone resorption activity. Collectively, these results indicate that 10^−7^–10^−6^M baicalin stimulated osteoclast maturation and function. However, high dose of baicalin (10^−5^M) repressed osteoclast maturation. This result is consistent with a previous study that demonstrated baicalin decreased osteoclast differentiation, since the lowest dosage in that study was 10^−5^ M. Mature osteoclasts are characterized by have multiple nuclei, an actin ring structure, and become acidic during bone resorption 18. All of these features are adapted for resorption of mineralized bone. The actin ring is required for osteoclasts to migrate to the active bone surface and initiation bone resorption 19. The bone resorptive activity of mature osteoclasts can be enhanced by development of an activated actin ring 20. We found baicalin enhanced production of actin ring which partially explain why it promoted osteoclast bone resorption activity. Expression of osteoclastogenesis marker genes including c‐Fos, c‐Src 21, NFATc1 22, 23, Fra‐1 24, MMP9 25, TRAP, CtsK and OSCAR was determined to be up‐regulated by baicalin and further confirmed our functional studies. However, the molecular mechanisms of baicalin bioactivity in regulating osteoclast maturation and function are still unknown.

Genetic evidence indicates that Mitf performed essential functions in osteoclast development 25. The bones of Mitf ^mi^/Mitf ^mi^ mutant mice showed the classic signs of osteopetrosis as a result of a lack of mature functional osteoclasts 26. In osteoclasts, Mitf has been shown to activate the expression of functional proteins TRAP, CtsK, OSCAR and E‐cadherin 27. TRAP is a metalloenzyme required for normal cartilage mineralization and adult bone remodelling. Cathepsin K is a cysteine protease important in the degradation of the bone organic matrix 21. OSCAR is a receptor for ECM collagens in osteoclasts and has been reported to be regulated by Mitf and NFATc1 28, 29. We suspect that Mitf might play a role in the molecular mechanisms that allow baicalin to regulate osteoclast maturation and function. Our data showed that baicalin significantly induced the expression of Mitf and enhanced its nuclear translocation. Furthermore, our immunofluorescent staining revealed that Mitf‐specific siRNA suppressed Mitf nuclear translocation induced by baicalin, which suggests that Mitf not only plays an important role in mediating baicalin effect on osteoclast differentiation, but also functions as a nuclear transcription factor during signalling transduction.

It is well established that the activation of MAP kinase plays a key role in RANKL‐induced osteoclast differentiation 30, 31, 32, 33. This induction results in the activation of Mitf, which is necessary for osteoclast differentiation and the control of osteoclast‐associated gene expressions 5. We showed simultaneous treatment with 10^−6^M of baicalin in the presence of RANKL remarkably enhanced osteoclast differentiation and maturation, which was mediated through p‐p38 and p‐ERK MAP kinase activations. Whereas baicalin alone e activated p‐ERK, so we employed a small molecule (U0126) that is a specific inhibitor of p‐ERK, to determine whether the enhancing effect of baicalin on osteoclasts can be blocked. We found U0126 abolished p‐ERK activation which inhibited osteoclast differentiation and maturation induced by baicalin. In addition, the expression of TRAP and other osteoclastogenesis genes induced by baicalin was also blocked by U0126 pre‐treatment. Baicalin‐induced Mitf nuclear translocation can be eliminated by U0126 treatment but silencing Mitf expression did not affect p‐ERK. This suggests that p‐ERK acts upstream of Mitf in the regulation of baicalin on osteoclast differentiation.

The receptor activator of nuclear factor kappa B (RANK) is located on the cell membrane of the osteoclast precursor cells. The binding of RANKL and RANK induces the monocytes to develop into the osteoclastic lineage. Activation of RANK results in the activation of downstream signalling transduction, binding of RANKL–RANK which triggers the NF‐κB and the MAPK pathways (including JUN, ERK, p38) 34. Ligand–protein prediction analysis has been used as a successful tool for fragment based drug discovery for the past 10 years 35, 36, 37. Small chemical structures are searched by the computer (such as silico system) for preferentially match to specific protein binding sites and this allows the discovery of the most potent binding site for small molecules in drug development 38, 39. In our ligand–protein prediction analysis, we determined that baicalin might dock on the Asn 262 residue of RANK side chains. It has been reported that at least 12 amino acids can form hydrogen bonds with the RANK side chains. Amongst these amino acids, Asn is one of the amino acids in ligand–protein binding. The distance between baicalin and Asn 262 is 2.3 Å (Fig. 6C). The distance between baicalin and RANK is within the required distance (2.0–3.4 Å) for forming hydrogen bonds. We hypothesize that the binding of baicalin to RANK can activate ERK/Mitf signalling which in turn promotes osteoclast maturation and function.

In conclusion, our study demonstrated that baicalin regulated osteoclast differentiation by activating the ERK/Mitf signalling pathway through the docking of baicalin to RANK. The findings contribute to a better understanding of the regulatory mechanisms of baicalin on osteoclast activities and offer an opportunity for developing a therapeutic drug to promote bone fracture healing and other associated bone diseases.

## Conflict of interest statement

All authors state that they have no conflicts of interest on this manuscript.

## Author contributions

Authors’ roles: LU Li, LI Qingnan and YANG Li designed the study; LU Li, RAO Li, JIA Huanhuan and CHEN Jun involved in data acquisition; LU Li, YANG Li, CHEN Jun, YANG Guozhu and LU Xingyan involved in data analysis and interpretation; LU Li drafted the manuscript; LU Li, LI Qingnan, LEE Ka Ho and YANG Li approved the final version of manuscript; and YANG Li take responsibility for the integrity of the data analysis.
